# Putative Pathobionts in HLA-B27-Associated Spondyloarthropathy

**DOI:** 10.3389/fimmu.2020.586494

**Published:** 2021-01-18

**Authors:** Tejpal Gill, James T. Rosenbaum

**Affiliations:** ^1^ Division of Arthritis and Rheumatic Diseases, Department of Medicine, Oregon Health & Science University, Portland, OR, United States; ^2^ Departments of Ophthalmology, Medicine, and Cell Biology, Oregon Health & Science University, Portland, OR, United States; ^3^ Legacy Devers Eye Institute, Portland, OR, United States

**Keywords:** pathobiont, HLA-B27, spondyloarthritis, gut inflammation, dysbiosis

## Abstract

Spondyloarthritis (SpA) is a group of immune mediated inflammatory diseases with a strong association to the major histocompatibility (MHC) class I molecule, HLA-B27. Although the association between HLA-B27 and AS has been known for almost 50 years, the mechanisms underlying disease pathogenesis are elusive. Over the years, three hypotheses have been proposed to explain HLA-B27 and disease association: 1) HLA B27 presents arthritogenic peptides and thus creates a pathological immune response; 2) HLA-B27 misfolding causes endoplasmic reticulum (ER) stress which activates the unfolded protein response (UPR); 3) HLA-B27 dimerizes on the cell surface and acts as a target for natural killer (NK) cells. None of these hypotheses explains SpA pathogenesis completely. Evidence supports the hypothesis that HLA-B27-related diseases have a microbial pathogenesis. In animal models of various SpAs, a germ-free environment abrogates disease development and colonizing these animals with gut commensal microbes can restore disease manifestations. The depth of microbial influence on SpA development has been realized due to our ability to characterize microbial communities in the gut using next-generation sequencing approaches. In this review, we will discuss various putative pathobionts in the pathogenesis of HLA-B27-associated diseases. We pursue whether a single pathobiont or a disruption of microbial community and function is associated with HLA-B27-related diseases. Furthermore, rather than a specific pathobiont, metabolic functions of various disease-associated microbes might be key. While the use of germ-free models of SpA have facilitated understanding the role of microbes in disease development, future studies with animal models that mimic diverse microbial communities instead of mono-colonization are indispensable. We discuss the causal mechanisms underlying disease pathogenesis including the role of these pathobionts on mucin degradation, mucosal adherence, and gut epithelial barrier disruption and inflammation. Finally, we review the various uses of microbes as therapeutic modalities including pre/probiotics, diet, microbial metabolites and fecal microbiota transplant. Unravelling these complex host-microbe interactions will lead to the development of new targets/therapies for alleviation of SpA and other HLA-B27 associated diseases.

## Introduction

**Figure 1:**
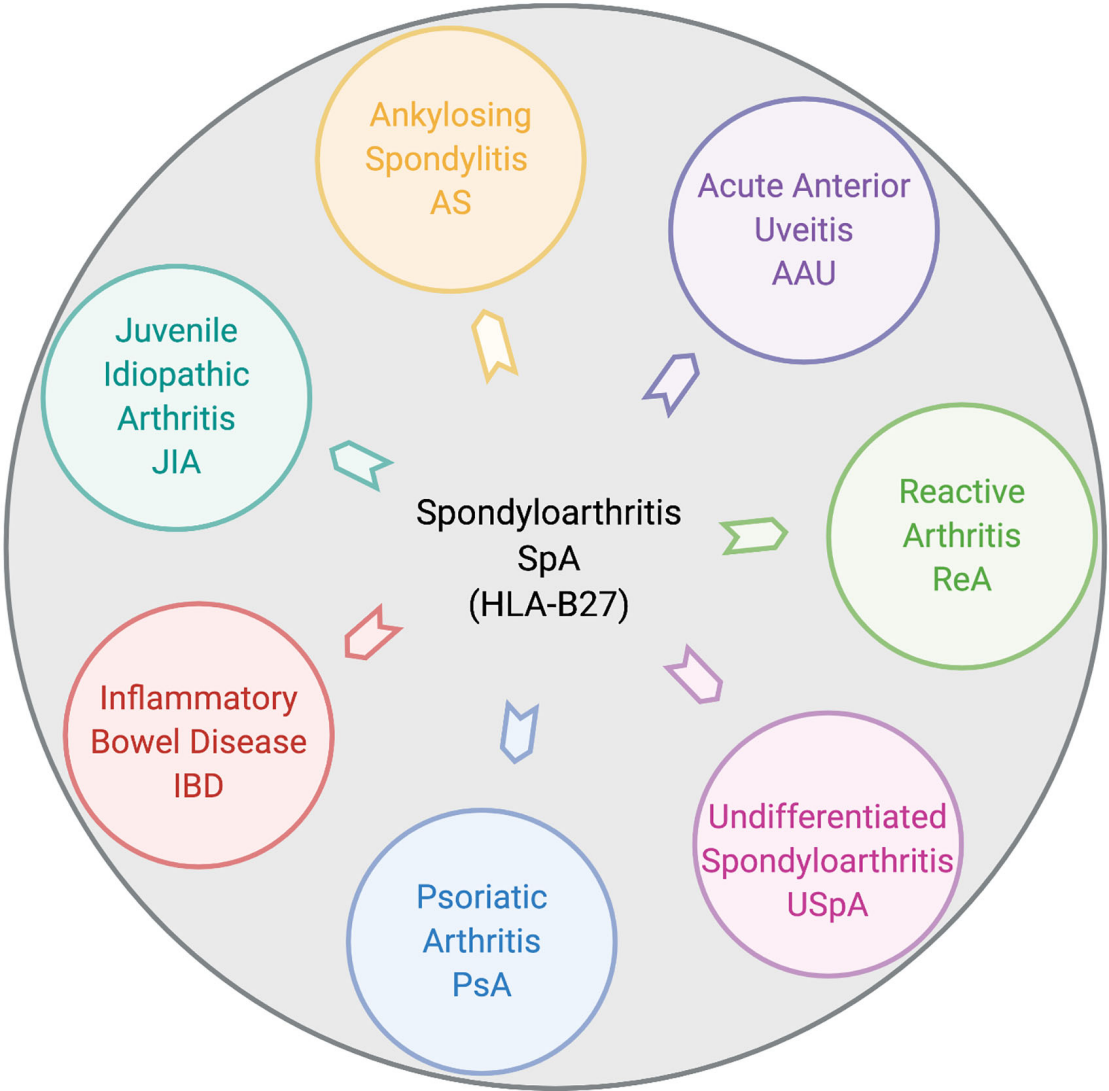
Disease overlap in HLA-B27-associated spondyloarthropathies. Pictorial representation of various disorders broadly included within HLA-B27-associated spondyloarthritis (SpA). These include ankylosing spondylitis (AS), acute anterior uveitis (AAU), reactive arthritis (ReA), juvenile idiopathic arthritis (JIA), inflammatory bowel disease (IBD), psoriatic arthritis (PsA), and undifferentiated spondyloarthritis (USpA). Figure created with Biorender.com.

**Figure 2:**
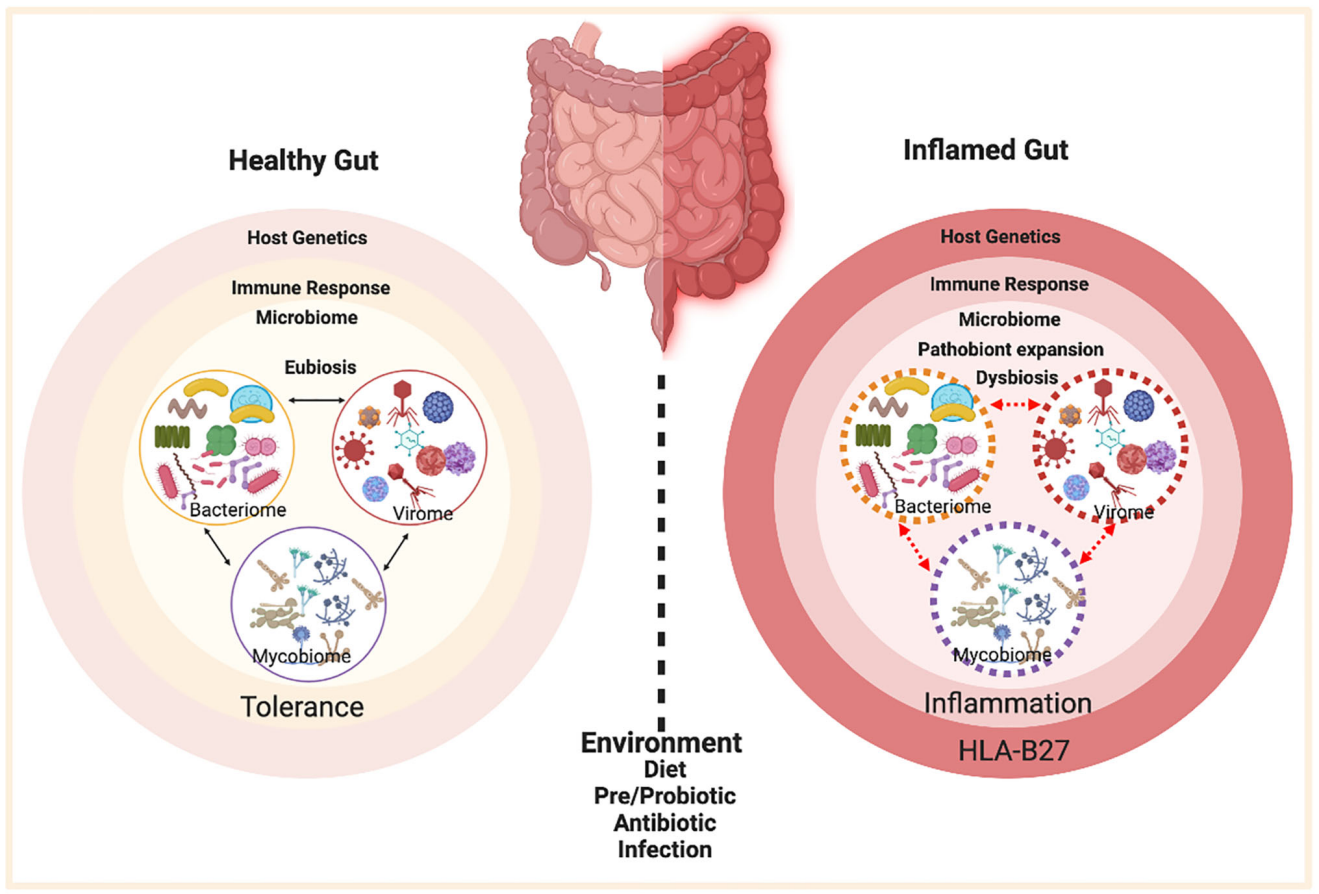
Factors contributing to HLA-B27 associated gut inflammation in SpA. In the healthy gut (left panel) there is microbial eubiosis/homeostasis within the members of bacteriome, mycobiome, and viruses in a stable interdependent microbial community. The gut immune response recognizes commensals and does not mount an inflammatory response. A non-susceptible host genetic background and a lack of environmental stress contribute toward favorable host-microbe interactions and microbial homeostasis. On the contrary, HLA-B27 associated gut (right panel) displays a microbial dysbiosis, which may be associated with pathobiont expansion, dysbiosis in various microbial components (bacteriome, mycobiome, virome) and loss of colonization resistance from the gut commensal microbes. HLA-B27 associated host immune dysregulation can cause loss of barrier protection and therefore bacterial components can activate an aberrant immune response, which in turn triggers an inflammatory response. Figure created with Biorender.com.

Spondyloarthritis (SpA) is an umbrella term used for various disorders including ankylosing spondylitis (AS), arthritis associated with inflammatory bowel disease (IBD), acute anterior uveitis, a subset of juvenile idiopathic arthritis (JIA), reactive arthritis (ReA), psoriatic arthritis (PsA), and undifferentiated spondyloarthritis (USpA) (**Figure 1**). These diseases share common clinical features (such as sacroiliitis, enthesitis and dactylitis) and overlapping extra-articular manifestations (i.e., uveitis, psoriasis, and bowel inflammation). Uveitis is the most common extra-articular manifestation of AS. In addition, many AS patients also have gut inflammation, such as Crohn’s disease (CD) and ulcerative colitis (UC). On the other hand, axial/peripheral arthritis is the most common extra-intestinal complications in IBD, especially in patients with CD (1, 2). These conditions may occur either simultaneously or sequentially, with almost 50% of AS patients having subclinical gut inflammation and around 15% of IBD patients have peripheral SpA (3, 4). In addition to similar and overlapping disease manifestation, there is a considerable overlap among the genetic risk factors for AS, CD, and PsA, such as *IL23R*, *IL12B*, *STAT3*, *ORMDL3*, and *CARD9* (5), which are associated with IL-23 signaling. The immune and inflammatory response between AS and CD shows considerable overlap dominated by the Th17 helper cell pathways (6, 7). In addition, association with a non-major histocompatibility gene Endoplasmic Reticulum Aminopeptidase 1 (ERAP1) has also been reported in patients with AS (8) and IBD (9) either alone or in combination with polymorphisms in HLA class-I alleles (10). Furthermore, other factors such as environment, host immune regulation, disruption of mucosal barrier, and gut microbial dysbiosis contribute toward pathogenesis of SpA [ (11, 12) **Figure 2**]. Host genetic susceptibility is associated with perturbed immune/inflammatory response, which may lead to microbial dysbiosis and pathobiont expansion and loss of barrier function, resulting in inflammation in the gut, joints, eye, and skin (13–16). While our focus for this review is on HLA-B27-associated microbes and their role in various SpAs, we will also discuss pathobionts and host-microbial relationships in IBD that are relevant to this topic.

## HLA-B27, Spondyloarthritis, and Gut Microbiome

Human leukocyte antigen (HLA)-B27, is a major histocompatibility complex (MHC)-class I molecule associated with various SpAs. Its association with the prototypic SpA- ankylosing spondylitis was discovered almost 50 years ago (17), and is the strongest association known between a genetic factor and a complex, genetic disease. Subsequently, the association of HLA-B27 was found with ReA, axial arthritis in association with IBD, AAU and the axial arthritis subset of PsA (18–20). The MHC complex is located on chromosome 6 (21), and plays a critical role in immunity and recognition of self in almost all cells of the body (22). MHC class I includes HLA-A, HLA-B, and HLA-C, and presents antigen to CD8 T cells. Despite HLA-B27 being a class I molecule, CD8 T-cells have not been associated with disease development; instead CD4+ T cells are thought to drive disease (23). While the actual role of HLA-B27 in triggering inflammation in various disorders remains unresolved, three different theories have been suggested. These include, i) presentation of arthritogenic peptides, which may activate a pathological immune response (24) ii) misfolding of heavy chain of HLA-B27 and its effects on ER-associated degradation (ERAD) and activation of unfolded protein response (UPR) (25–28); and iii) HLA-B27 dimerization during cell surface recycling and recognition by immune receptors on natural killer (NK) cells (29, 30). While these hypotheses may provide some explanation, such as the activation of the inflammatory IL-23/lL-17 axis through UPR or non-canonical activation of CD4+ T cells by HLA-B27 dimers, the exact mechanism by which HLA-B27 leads to development of SpA is unknown (31). In the last decade, HLA-B27-associated perturbation in the gut microbiota has emerged as an underlying mechanism in disease pathogenesis. Indeed, many of the risk genes common to IBD and SpA (e.g., *IL23R*, *NOD2*) are associated with innate immune response pathways (32, 33), consistent with the hypothesis that alteration in the host immune response to gut microbes may play a key role in these disorders.

More than twenty-five years ago, a new insight came from the experimental data revealing the role of gut microbiota in complex inflammatory disorders. In 1994, it was observed that HLA-B27 transgenic (TG) rats raised in a germ-free environment fail to develop either arthritis or colitis (34). Recolonizing the gut with either altered Schaedler’s flora (ASF) or just a few commensal microbes was sufficient for disease development (35, 36). Rosenbaum and Davey proposed a hypothesis that ‘HLA-B27 predisposes to ankylosing spondylitis by altering the microbiome’ (37). Since the last decade many studies in patients and rodent models have shown alteration of gut microbial communities in various HLA-B27 associated disorders (12, 38–40). Based on our extensive studies with the HLA-B27 TG rat model, we demonstrated that HLA-B27-associated microbial dysbiosis is subject to host genetics and environment. This lead to the proposal of an ecological model of microbial dysbiosis (12).

## Putative Pathobionts in hla-b27 Associated Spondyloarthropathies

Through the advent of high throughput sequencing approaches, many putative pathobionts have been identified in various HLA-B27-associated SpAs and bowel inflammation. A pathobiont is defined as a microbe that can cause or promote disease only when specific genetic or environmental conditions are altered in the host such as dysregulated host immune response and microbial dysbiosis (41). The term “pathobiont” was first used for *Helicobacter hepaticus*, a bacterium associated with gut inflammation in immunocompromized mice, but not in wild-type animals (42, 43). While most of the sequencing studies have focused on the alterations in bacterial component of the gut microbiota, recent research is also focused to define the fungal and viral component of the gut microbiome (mycobiome and virome respectively) and interkingdom interactions in SpA. In this section, we will discuss important bacterial, fungal, and viral pathobionts in HLA-B27-associated SpAs including overlapping inflammatory diseases such as CD and colitis.

### Bacterial Pathobionts

#### Pathobionts Associated With Reactive Arthritis

Reactive arthritis (ReA) is an immune mediated inflammation of the synovial tissue that usually develops after a urinary or gut infection (44). HLA-B27-associated ReA is a type of SpA triggered by bacteria such as *Campylobacter*, *Chlamydia*, *Salmonella*, *Shigella*, and *Yersinia*, resulting in oligoarthritis of the lower limbs and sometimes with urethritis and conjunctivitis (45). One of the earlier studies isolated *Chlamydia trachomatis* strains from the eye and urethra of a patient with ReA, previously known as Reiter’s Syndrome (46). Subsequently, in a study focused on endoplasmic reticulum (ER) stress, which is thought to be downstream to HLA-B27 protein misfolding, *C. trachomatis* was shown to induce IL-23 expression in infected myeloid cells (47). In addition, stimulation through TLR or ER stress can cause the activation of ER stress induced transcription factor CHOP, which in turn increases the expression of IL-23. However, TLR engagement in itself can trigger ER stress through activation of XBP1, essential for production of inflammatory cytokines in macrophages (48). HLA-B27 misfolding was associated with enhanced replication of *Salmonella* by the activation of the unfolded protein response (UPR), through the transcription factor XBP1 (49) Although this study employs HeLa cells that do not express TLR, it is possible that innate immune receptors may be involved in response to bacteria or bacterial products (50). In another study, peripheral blood and serum polymerase chain reaction (PCR) analysis in patients with *Chlamydia* induced ReA showed the presence of DNA from *C. trachomatis* in the peripheral blood cells, but not in the serum (51). This can be explained by the fact that *C. trachomatis* resides in the monocytic cells, prevents their apoptosis and stimulates the production of inflammatory mediators (51). In fact, it is a hallmark of Chlamydia induced ReA, in which bacteria causing synovitis persist in low quantities making it hard to detect using PCR or culture techniques. However, Freise and colleagues later standardized PCR detection for *C. trachomatis* from synovial fluid (52). This may explain why various attempts to cultivate other pathogenic bacteria such as *Yersinia* or *Salmonella* from affected joints have yielded negative results (53, 54). Instead, *Salmonella* and *Yersinia* antigens have been identified in synovial fluid and/or tissue by immunohistochemistry. Antibodies against LPS from these microbes has been shown to be present in the synovial fluid many years after the infection which explains the strong IgA responses in people who have had a bout of ReA (53, 54). In a mouse model of *Salmonella enteritis* induced joint inflammation, increased levels of inflammatory cytokines IL-17 and TNF-α were observed in the mesenteric lymph node and synovium respectively. Neutralizing IL-17 in mice infected with *S. enteritis*, prevented synovitis and curbed the increase in TNF-α, suggesting the role of IL-17 in gut and joint inflammation (55). In addition to these pathogenic microbes, diarrheagenic *Escherichia coli* (DEC) has also shown to increase the incidence of musculoskeletal symptoms in individuals who contracted DEC associated diarrhea during their international travels. Of these patients, a small number of patients also met the criteria for ReA (56). Thus, while ReA may be highly associated with various bacterial enteric pathogens such as *Campylobacter* and *Salmonella* (57), its incidence after DEC infections is low. Recent study in the *Yersinia enterocolitica* murine model of ReA (*TNFRp55-/-* mice) has shown an important role for mesenteric dendritic cells. Intestinal dendritic cells migrated to the regional lymph nodes and contributed toward the immunopathogenesis of ReA (58). It is important to note that in most cases, infections with enteric pathogens do not result in development of ReA. In a comprehensive review by Ajene and colleagues (59), the ReA incidence for *Salmonella*, *Campylobacter*, and *Shigella* ranged from 0.1%–29%, 0%–16%, and 0%–12% respectively. Nevertheless, in studies in which enteric pathogens do lead to the development of ReA, the ability of a bacterium or bacterial antigen to reach the joint or gain access to particular cells such as the macrophages and evade the host defense might play an important role. In view of these different microbes associated with ReA, disease pathogenesis is thought to involve host-microbe interactions as evident with the presence of bacteria or their products in the joint, followed by local immune response.

#### Altered Schaedler’s Flora (ASF)

In 1965, Russell Schaedler developed a model microbial community to colonize germ-free animals to prevent the colonization of opportunistic pathogens (60), which was later modified to be more representative of gut microbiota and renamed as Altered Schaedler’s Flora (ASF) (61). 16s rRNA sequencing of ASF was performed to define the phylogeny of the ASF microbes (namely two members of *Clostridium* sp., *Lactobacillus intestinalis*, *Lactobacillus murinus*, *Mucispirillum schaedleri*, *Eubacterium plexicaudatum*, *Pseudoflavonifractor* sp., and *Parabacteroides goldsteinii)* (62, 63). Even though the ASF is a reductionist model microbial community, functional analysis of the ASF metagenome compared with the wild mice metagenome showed the functional similarity between gut microbiome of ASF and wild type mice (64). Early experiments colonizing germ-free HLA-B27 TG rats with ASF played a pivotal role in establishing the role of gut microbiota (especially *Bacteroides*) in the development of gut inflammation (35). Another mouse model for SpA is the SKG model, which has a mutation in the ZAP-70 (T cell receptor signaling gene). Upon injecting with curdlan, a component of bacterial and fungal cell walls, these mice develop SpA with uveitis, arthritis, and CD like ileitis (65, 66). Germ-free SKG mice recolonized with ASF exhibit increased arthritis incidence, although the severity of arthritis was attenuated in comparison with the specific pathogen free (SPF) mice (66). SPF mice had the highest incidence of Ileitis followed by ASF recolonized mice, while the germ-free mice did not have Ileitis (66). This suggests that dysregulation of mucosal-microbe interface is necessary for the development of ileitis, which fails to occur in germ-free SKG mice. In comparison, ileitis is mild in SKG mice colonized with ASF, highlighting the importance of a diverse microbial community in disease development. ASF studies have paved a way to understand the host-microbe interaction in a measurable way and have emphasized the role of commensal microbes acting as pathobionts in disease development.

#### *Dialister*

*Dialister* is a saccharolytic bacteria, belongs to family *Vellionelaceae* (67), that can convert succinate to propionate (68). Tito and coworkers (69) studied the relationship between the intestinal microbial composition of ileal and colon biopsies from inflamed and non-inflamed tissues, and observed SpA-associated microbial dysbiosis. Of note, they found that *Dialister* was increased in the inflamed tissue and positively correlates with the disease score, whereas the non-inflamed tissue had low frequency of *Dialister*. Another study examining post-infectious SpA reported an increase in the relative abundance of *Dialister*. Subjects who developed enthesitis also had increased abundance of *Campylobacter* and subjects with uveitis and radiographic sacroiliitis had increased abundance of *Erwinia* and unclassified *Ruminococcaceae*, respectively (70). Interestingly, some species of *Dialister* such as *D. pneumosintes* and *D. invisus* are shown to be pathogenic in orthodontic infections (71). Oral pathobionts can colonize the gut during inflammation, as there is increased availability of oxygen and lack of colonization resistance during inflammatory conditions (72). This in turn drives the Th1 response primarily by interferon gamma (IFNγ), and exacerbates gut inflammation (72). Another study focused on microbial dysbiosis associated with HLA alleles in healthy subjects with AS, and rheumatoid arthritis (RA), a chronic autoimmune disease defined by inflammation of the synovium and joint destruction (73). However, the authors did not observe HLA-B27-associated changes in *Dialister* in healthy subjects with either AS or RA associated alleles. While one study focused on the microbiota from biopsies collected from post-infectious SpA patients (69), the latter study focused on the fecal samples from HLA-B27 positive healthy individuals, many of whom will not develop disease. Stated differently, while it is possible for patient cohorts from different geographical locations to have distinct microbes driving disease, differences due to sampling location and disease severity also contributes to the association with distinct pathobionts.

#### *Blautia*

Another pathobiont associated with AS is *Blautia*. It is a member of the family *Lachnospiraceae*, which has been associated with gut inflammation in an experimental model of SpA (48, 74). A recent study by Zhang and colleagues (75) on the fecal samples from AS patients in a Chinese cohort has shown *Blautia*, *Megamonas* and *Dorea* associated with AS patients with a concomitant decrease in *Lachnospira*, *Ruminococcus*, and *Clostridium_XlVb*. In a study on patients with ReA, the authors reported an increase in enteropathogens such as *Erwinia* and *Pseudomonas* as well as several other microbes including *Blautia*, *Coprococcus*, *Roseburia*, and *Collinsella* (70). Many of these microbes (e.g., *Blautia*) were thought to be gut commensals, but new studies have shown them to be increased specifically with disease and thus a pathobiont. In a rat model of SpA, *Blautia* has been associated with HLA-B27 and SpA on the Lewis background, but not on the Fischer background (12). Hablot and colleagues (76) compared the microbial dysbiosis in mice with dextran sodium sulfate (DSS) induced colitis from mice with arthritis and colitis (induced with collagen and DSS). They found that mice with arthritis and colitis had increased relative abundance of *Blautia*, *Gemellaceae*, and *Ruminococcus gnavus* as compared with the colitis only group. Both *Blautia* and *Ruminococcus* are closely associated members of the family Lachnospiraceae, which are among the main producers of short chain fatty acids (SCFAs), and many taxa of this family are associated with various inflammatory diseases (77). While the role of *Blautia* in HLA-B27 associated SpAs was discussed in this section, the role of *Ruminococcus gnavus* as a pathobiont is discussed below.

#### *Ruminococcus gnavus*

*Ruminococcus gnavus* is a known pathobiont associated with SpA and associated IBD (51, 78). In a cohort of SpA patients and related as well as unrelated healthy controls, there was an increase in the relative abundance of *R. gnavus*, which correlated with the disease activity and with patients having a history of IBD. This change was not observed in their subjects with RA (78). Increases in *R. gnavus* have been associated with other inflammatory diseases including inflammatory bowel disease (79, 80), CD (81), and pouchitis in UC patients (82). Another instance of increased abundance of *R. gnavus* comes from patients with systemic lupus erythematosus (SLE), an autoimmune disease characterized by a hyperactive immune system which causes inflammation in many tissues, as well as an aberrant antibody response. Most SLE patients will develop either arthritis or synovitis sometime during their disease. A study on SLE patients showed an increased amounts of *R. gnavus* that correlated with disease activity, which was highest in the patients with lupus nephritis (83–85). One plausible mechanism for the contribution of *R. gnavus* in an inflammatory disease, CD, has been shown by Henke and colleagues (86). They found that *R. gnavus* secretes a complex gluco-rhamnan polysaccharide, which can induce the production of inflammatory cytokines like TNFα by activation of TLR4 on the dendritic cells and may explain the mechanism underlying the association between gut inflammation in CD and *R. gnavus*. TNFα is a potent inflammatory mediator in both SpA and IBD. This may explain why an increase in the relative abundance of *R. gnavus* in patients correlates with disease severity in both arthritis and gut inflammation. *R. gnavus* is also shown to provide colonization resistance to the gut microbial community by the production of bacteriocin ruminococcin A, which is active against pathogenic members of class *Clostridia*, especially *Clostridium perfringens, Clostridium difficile*, and other members phylogenetically related with *R. gnavus* (87), thus providing a competitive edge in gut colonization and inflammation. These studies show the harmful role of R. gnavus through different mechanisms. Association of *R. gnavus* and other pathobionts with both SpA and IBD may partially explain the overlap between the mechanisms underlying these complex inflammatory diseases in many patients.

#### *Akkermansia muciniphila*

Many studies on human and animal models of SpA have shown increased abundance of *Akkermansia muciniphila*, a mucin degrading bacteria found in human intestinal content (88). A study on pediatric SpA cohort, the microbiota from patients separated from healthy controls, and was divided subjects into two clusters each dominated by increased levels of either *A. muciniphila* or genus *Bacteroides* (89). Furthermore, to evaluate the pathogenicity of altered microbial composition in children with SpA, the group performed fecal microbial transplant to germ-free K/BxN mice. Transplanted mice displayed over-representation of *Bacteroides* and *Akkermansia*, and the latter positively correlated with disease activity. Addition of *Akkermansia* to ASF also increased the permissiveness to arthritis in these mice, when compared to mice that received ASF alone (90). Metagenomic analysis of fecal samples from patients with enthesitis related arthritis (ERA) showed decrease in the relative abundance of *Faecalibacterium prausnitzii* in both pediatric and adult SpA cohort, while the relative frequency of *Bacteroides fragilis* was increased in pediatric SpA cohort and decreased in adult SpA cohort (91). *A. muciniphila* has also been associated with disease in experimental SpA. In HLA-B27 TG Fischer rats, the relative abundance of *A. muciniphila* was increased along with elevated IgA coating of intestinal microbes (11). In a subsequent study, we compared the effect of host genetic background on microbial dysbiosis and found that the increased level of *A. muciniphila* was found in the Fischer HLA-B27 rats in comparison to Fischer wild-type controls; while in Lewis HLA-B27 TG rats there was an increase in the relative abundance of *Prevotella* when compared to Lewis wild type controls. Addition of *A. muciniphila* in germ-free and SPF *IL10-/-* mice is sufficient to exacerbate gut inflammation. IL10 is an anti-inflammatory cytokine and *IL10-/-* mice develop chronic colitis with marked increase in pathological type-I helper T cell response (92) in SPF but not in germ-free conditions. NLRP6 deficiency in these mice results in the enrichment of *A. muciniphila*, which then acts as a pathobiont in the development of colitis (93), and highlights the ability of NLRP6 in regulating colonization of colitogenic bacteria. Contrary to the role of *A. muciniphila* in inflammation, relative abundance of *A. muciniphila* has been shown to have an inverse correlation with obesity and metabolic diseases (94, 95). In fact, supplementation of *A. muciniphila* reversed high fat diet induced obesity in mice, which was mediated by altered adipocyte metabolism and improved gut barrier function (96). *Akkermansia* is a short chain fatty acid producer (97) and is thought to increase fatty acid oxidation in intestines and adipose tissues (98). Taken together, these distinct and opposite (pathobiont vs commensal) roles of *A. muciniphila* in various disorders highlight the tight regulation of the microbial abundance within the community and the effect of dysbiosis (increase or decrease in relative abundance) in health and disease.

#### *Prevotella*

*Prevotella* is another mucus degrading pathobiont that shows gut inflammation associated increased relative abundance in *NLRP6-/-* mice (99). *Prevotella* can reach to the crypt in the mucosal layer of the gastrointestinal tract, and are associated with SpA. Metagenomic analysis from the gut microbial DNA from a Chinese cohort of AS patients has revealed the abundance of Prevotella *melaninogenica, Prevotella copri*, and *Prevotella* spp. C561 and decreased abundance in *Bacteroides* spp (100). Interestingly, they also observed increased abundance of the *Bifidobacterium* genus, a commensal gut bacteria commonly found in probiotics. On the contrary, there was a decrease in the abundance of family *Prevotellaceae* in AS patients. This may be due to differences in the cohorts (Chinese vs caucasian), sampling location (fecal vs ileal biopsy), or it could also be due to the relationship between other members of the microbial community and/or the effect of host genetics (100). Scher and colleagues observed increased relative abundance of *P. copri* has also been reported in 16s microbiome sequencing of fecal samples from patients with new onset RA (101). They performed metagenomic sequencing of patient derived *Prevotella* strains and also compared the metagenomes between healthy controls and new-onset RA patients. Patients with new onset RA had decreased abundance of vitamin metabolism (i.e., biotin, pyroxidal, and folate) and pentose phosphate pathway which was consistent with Prevotella genomes lacking these functions. To determine whether Prevotella copri was sufficient to drive gut inflammation, they gavaged antibiotic treated mice with P- copri and after 2 weeks they found that P. copri had dominated the gut microbiota in these mice and exacerbated the susceptibility to DSS induced colitis (101). Consistent with these mouse studies, another study found enrichment of *P. copri* in patients during the pre-clinical phase of RA, before disease onset (102), which suggests a role of *P. copri* in intestinal dysbiosis and disease susceptibility. A recent cross-sectional study utilized data from a previous TwinsUK cohort, and used genotyping and microbiota data after excluding patients with RA and their twins. The authors found *Prevotella* spp in the gut microbiota of individuals who had RA associated genotype associated without the disease. again suggesting a role for host-microbe interactions prior to disease onset (103).

#### *Mucispirillum schaedleri*

*Mucispirillum schaedleri*, a Gram negative pathobiont, is a member of ASF, and is known to colonize the gut mucus layer in rodents (104). It was reported that in mice having a combined deficiency of two susceptibility genes for CD, namely nucleotide-binding oligomerization domain-containing protein 2 (NOD2) and NADPH oxidase, disease can be induced by *M. schaedleri* (105). *NOD2* pays an important role in microbial regulation in the ileum (106), whereas *NADPH oxidase* is known to regulate the gut intestinal barrier through the production of reactive oxygen species (107). The authors demonstrated that in the absence of both *NOD2* and phagocytic *NADPH oxidase*, there is accumulation of *M. schaedleri* in the gut lumen and mucosa associated with gut inflammation. Since, *Mucispirillum* is a bacterium found in rodents, it is an unlikely contributor to human diseases. However another bacterial taxa, *Proteobacteria*, which is closely related to *Mucispirillum* have been reported to have increased abundance in both patients with SpA (108) and CD (109). In contrast, another study reported that *M. schaedleri* can protect mice against *Salmonella typhimurium* virulence factors (110). This was supported by data from our study with the HLA-B27 TG rats, in which we found a decrease in the relative abundance of *M. schaedleri* as compared to the wild type rats (12), which may suggest a protective effect of the microbe. Since M. *schaedleri* is a mucolytic bacterium like *A. muciniphila*, we can hypothesize that it may be beneficial to the host at low relative abundance within the microbial community. However, increased abundance of *M. schaedleri* may compromise the spatial segregation by bringing luminal microbes close to the intestinal epithelial cells, thereby triggering an inflammatory response.

#### *Adherent Invasive Escherichia coli*

Invasive properties of various bacteria such as *E. coli*, *S. typhimurium*, and *Citrobacter rodentium*, may be critical for their ability to colonize the host. Of these, *E coli* has been associated with the induction of gut inflammation in CD (111). A study characterizing adherent invasive *Escherichia coli* (AIEC) found that these *E. coli* may harbor genes associated with bacterial adhesion and invasion and therefore regulate barrier permeability contributing to gut inflammation (112). Another study assessed the prevalence of AIEC associated with the intestinal mucosa of patients with CD, UC, and of healthy controls (113). They found that AIEC was found associated with the inflamed regions in the ileal mucosa in patients with CD, but was not observed in the ileal mucosa of patients with UC. In another study on the IgA coated bacterial fraction from patients with CD-associated SpA detected enrichment of *E. coli* in comparison to patients with CD alone. This IgA coated *E. coli* fraction displayed genotypic and phenotypic similarities to AIEC. Colonization of these AIEC in germ-free mice induced inflammatory Th17 mucosal immune response in comparison to colonization with non-AIEC strains of *E. coli* (114). This study identified immune reactive pathobionts that provide a link between mucosal immunity and systemic inflammation in CD-associated SpA and may guide future therapies.

### Fungal Pathobionts

While microbial dysbiosis and pathobiont enrichment have been associated with SpA and associated CD for almost two decades, most of the work has been focused on the gut bacteria and some *Archaea*. However, fungal products such as β-glucan have been known to trigger SpA and ileal inflammation in SKG mice model (BALB/c ZAP-70W163C mutant) of SpA (115). Another study has shown the association of anti-*Saccharomyces cerevisiae* antibodies (ASCA) with intestinal inflammation in patients with Ax SpA and associated CD (116). In the last decade, with new and advanced approaches to characterize the fungal component of the gut microbiome (mycobiome), their role in disease pathogenesis is being studied more extensively. In a recent study in patients with SpA, treatment with IL-17 inhibitors was associated with a shift in bacterial and fungal taxa, specifically the bacteria from the family Clostridiales and the yeast *Candida albicans* (117). In these patients, the changes in the gut microbiome were associated with the perturbations in metabolic pathways and overexpression of IL-17/23 cytokines and the expansion of IL-25/17 producing tuft cells as well as type 2 innate lymphoid cells (ILC2), both of which are implicated in helminth immunity (117). A study investigated the mycobiome in IBD (CD and UC) and found increased intestinal fungal diversity in patients with CD in comparison with healthy controls (118). However, they did not observe any difference between the fungal species between the CD and UC groups. Another study focused on the relationship between *Candida albicans* and gut inflammation by using mice that lack Galectin 3 (Gal3-/-), an intestinal lectin that binds specifically to C. albicans, and showed that in Gal3-/- mice, DSS colitis was worse in comparison to wild type mice, with enhanced colonization by *C. albicans* (119). This revealed the role of *C. albicans* in augmenting DSS mediated colitis as well as the role of Gal-3 in preventing colonization by *C. albicans*. In another example of host fungal interaction, Iliev’s group (120) illustrated that the fungal community in the gut interacts with the immune system through innate immune receptor Dectin-1. In the DSS colitis model, mice deficient in Dec-1 had exacerbated colitis if challenged with *Candida tropicalis* whereas WT mice did not show an increase in colitis. In a study focused on both micro- and myco-biota in AS patients, the authors showed an increase in the levels of *Ascomycota*, where altered mycobiota was associated with the degree of radiographic damage (121). El Mouzan and colleagues (122, 123) investigated the gut fungi in treatment naive new onset CD in a pediatric Saudi Arabian cohort and found fungal dysbiosis associated with CD patients without the loss of fungal diversity between CD patients and healthy controls (HCs). They found that patients with CD had an increase in *Psathyrellaceae, Cortinariaceae, Psathyrella*, and *Gymnopilus* with a concomitant decrease in *Monilinia*. In a recent study, the authors found *Malassezia restricta*, a common skin commensal fungus, associated with the intestinal mucosa in CD patients (124). *M. restricta* was specifically associated with individuals carrying the IBD risk gene, CARD9-, a signaling adaptor protein with an antifungal role. The study showed that CARD9 variants present in these patients can induce the host immune cells to produce inflammatory cytokines against *M. restricta*. In a mouse model, *M. restricta* exacerbated colitis in germ-free as well as gnotobiotic mice. Taken together, these studies display the importance of fungi in HLA-B27-associated SpAs and highlight the importance of host-bacteria-fungal interactions in these diseases.

#### Fungi-Bacteria Functional Interaction

Recent studies have shown the role of inter-kingdom fungal-bacterial interactions contributing to various SpAs. One such study showed investigated the bacterial-fungal interkingdom networks in AS patients (121) and showed perturbed relations between gut bacteria and fungi as evident by decreased fungal to bacterial biodiversity ratios in these patients. Another study looking into the mycobiome of Japanese CD patients showed an increase in the abundance of *C. albicans*, *Entyloma*, and *Trichosporon* in the CD patients in comparison with the HCs. In contrast the HCs had increased abundance of *Saccharomyces* and *Sarocladium* in comparison to the CD patients. Microbial dysbiosis was also observed in CD patients as evident by decreased microbial diversity and increased abundance of *Enterococcus* in CD patients (125). Bacterial-fungi correlations showed positive correlation between *Enterococcus* and *Malassezia*. CD patients showed a positive correlation between *Ruminococcus* and *Sarocladium* and *Ustilago* (125). These associations are especially interesting as these bacteria and fungi have been separately associated with various spondyloarthropathies. In another study, it was shown that patients with CD are associated with increased levels of the fungus *Candida tropicalis* and two bacteria *E. coli* and *Serratia marcescens. C. tropicalis* positively correlated with *E. coli* and *S. marcescens* in these patients and was observed to associate closely in biofilms in comparison to other microbes (126). Studies are also investigating specific bacteria-fungi relationships in rodent models. Mice treated with DSS to induce colitis showed an increase in disease severity when supplemented with *Candida albicans*. On the other hand, colitis improved in these mice with the addition of *Saccharomyces boulardii*. Treatment with antibiotics affected the disease severity and the effects of fungi on colitis. While treatment with vancomycin that targets all Gram negative microbes protected mice from colitis, treatment with colistin to target *Enterobacteraceae* specifically retained the colitis phenotype (127). Disease was not affected by addition of either *C. albicans* or *S. boulardii*. Fungal-bacterial correlations were decreased severely in the colistin treated mice, suggesting that effect of fungi on colitis was due to its interaction with bacteria belonging to family *Enterobacteraceae*. Restoring the *Enterobacteraceae* in these mice restored the effect of both *C. albicans* and *S. boulardii* on the colitis (127). These studies highlight that microbial functions are considerably affected by various positive and negative trans-kingdom interactions between the members of the bacteria and fungal community.

### Viral Pathobionts

The human gut virome is another emerging component of the gut microbiome, which is thought to impact human health either directly or *via* the modulation of the bacteriome through bacteriophages. However, the virome has been more of a dark matter with studies on a limited number of known viruses. A recent study by Norman and others (128) showed that the virome was altered in IBD with a significant expansion of *Caudovirales* bacteriophages. They compared the bacterial and *Caudovirales* bacteriophage communities and found distinct relationships in CD and UC patients. It was associated with increased richness but decreased viral diversity and these changes were concomitant to the changes in the bacterial communities suggesting a role of viral perturbations in IBD. Studies on the human virome have shown that viruses display bacteria like inter-individual variability and also respond dynamically to various environmental influences like diet (129, 130). In the healthy gut the viral core is made of virulent phages. However, in patients with CD, the virome shifts toward a temperate viral core and the changes in the viral community affect the bacterial community (131). Determining the virome in HLA-B27 mediated diseases may shed light on pathogenesis and may be crucial for the development of phage biomarkers.

So far, we have focused on specific bacterial, fungal, and viral pathobionts associated with HLA-B27-assocated SpA and other immune/inflammatory disorders that overlap clinically with SpA. However, determination of disease associated microbes may also depend upon other environmental factors such as geographical location, diet, genetic factors (other than HLA-B27), as well as technical factors like sampling location, related vs unrelated controls, methods of sequencing and data analysis. Spatial heterogeneity of microbial community profile through the gastrointestinal tract has shown to vary immensely. For example, the fecal microbiota provides a view of the microbial diversity at a given time point, and is used in majority of the microbiome studies, it neglects the mucosa-associated microbes (132). In addition, effects of related and unrelated healthy controls along with related healthy controls that cohabit need to be considered, as they can introduce variability. While efforts are being made to standardize the microbiome studies (133), attention toward the host, environmental and technical difference issues will be highly valuable while inferring results from multiple studies.

## Pathobionts vs Dysbiosis

The gut microbial community is diverse with enormous inter-individual variability due to host genetics and other environmental factors, which may explain why different studies on SpA with diverse patient cohorts have reported expansion of distinct disease-associated microbes or pathobionts. In healthy individuals, pathobionts are present in relatively low abundance and increase during dysbiosis in disease susceptible individuals, contributing to pathogenesis. This could be an active increase in their relative abundance due to changes in their microenvironment, or they can increase as the colonization pressure from gut commensals is lost due to inflammation. Therefore, it is vital to study these pathobionts in the context of their host genetics and microbial community structure. IL-2 knockout mice have dysregulated T cell functions and develop chronic immune mediated colitis in SPF mice (134), however these mice like the *IL-10-/-* mice discussed earlier fail to develop colitis under germ-free conditions (135). These studies emphasize the interaction between the host genetics and gut microbiome in disease development. This is exemplified in HLA-B27 rats, which also fail to develop colitis in germ-free conditions. Introduction of *Bacteroides vulgatus* in germ-free HLA-B27 TG rats is sufficient to induce colitis, but its introduction in SPF raised athymic HLA-B27-TG rats fails to induce colitis (136). In contrast, addition of *B. vulgatus* is not sufficient to induce colitis in *IL-10-/-* mice, which suggested that resident enteric bacteria are necessary for immune activation and this development of spontaneous colitis in this model. In fact, presence of *B. vulgatus* protects the *IL-2-/-* mice from developing *E. coli* induced colitis, which underscores that different microbes and their interactions may dictate their ability to trigger disease (36, 137). In *TNF^ΔARE^* mice model, biosynthesis of TNF is dysregulated, leading to the development of chronic inflammatory arthritis and CD like ileitis. Germ-free *TNF^ΔARE^* mice did not develop ileitis, even when colonized by a pathobiont *E. coli* LF82. Development of CD like gut inflammation occurs only when these mice were colonized with cecal content from inflamed mice raised in SPF condition (138). Collectively, these studies performed with different genetic susceptibility models pinpoint the joint role of diverse microbiota and genetic susceptibility as a requirement for development of SpA or associated gut inflammation.

Metchnikoff (1908) observed that intestinal microbes are dependent on our dietary intake, and therefore it may be possible to modify the gut microbiota. He defined “dysbiosis” as ecological imbalance in the gut microbial community. While the concept of microbial dysbiosis was forgotten in the following decades with the focus on antibiotics, and lack of ability to culture and classify gut microbes, which are mostly obligate anaerobes and refractory to culture. Recent advances in culture free determination of microbial community members using next generation sequencing abilities, the microbial dysbiosis and its role in human health and disease has been a focus. These studies have classified many distinct features of microbial dysbiosis, such as reduced microbial diversity, pathobiont expansion and loss/alteration of microbial community structure (139). Another important feature of microbial dysbiosis is the perturbation in the metabolic function, which has been associated with many immune and inflammatory disorders including IBD and SpA (139–142).

In our study with the HLA-B27 transgene (12) on three different rat genetic backgrounds namely Lewis, Fischer and Dark agouti (DA), we have demonstrated that the gut microbiome is dependent on the host genetics and environment. Only two of the backgrounds (Lewis and Fischer) were disease susceptible, whereas the DA rats were resistant to HLA-B27-associated SpA. In disease susceptible Lewis and Fischer backgrounds, HLA-B27-associated gut microbial dysbiosis was dependent on the host background, while the immune dysregulation was independent of the host genetics and environmental effects, and showed considerable overlap of inflammatory mediators between HLA-B27 TG Lewis and HLA-B27 TG Fischer rats. Analyzing the predictive metabolome and host-microbe inter-omic analysis suggested that in comparison with their respective wild type controls, HLA-B27-associated with different pathobionts in Lewis (*Prevotella*) and Fischer (*Akkermansia*, members of family *Lachnospiraceae*). However, the microbial functional/metabolic pathways perturbed in both backgrounds were similar. In both these backgrounds, these diverse pathobionts are associated with common host genes for immune/inflammatory pathways (74). This led us to propose an ecological model of dysbiosis where perturbation of the microbial community structure and function contributes to disease pathogenesis, instead of a single microbe driving disease (12). These studies suggest that gut microbial functions are highly dependent on their community structure and the gut microenvironment (143).

### Pathobionts Are Context Dependent

**Figure 3:**
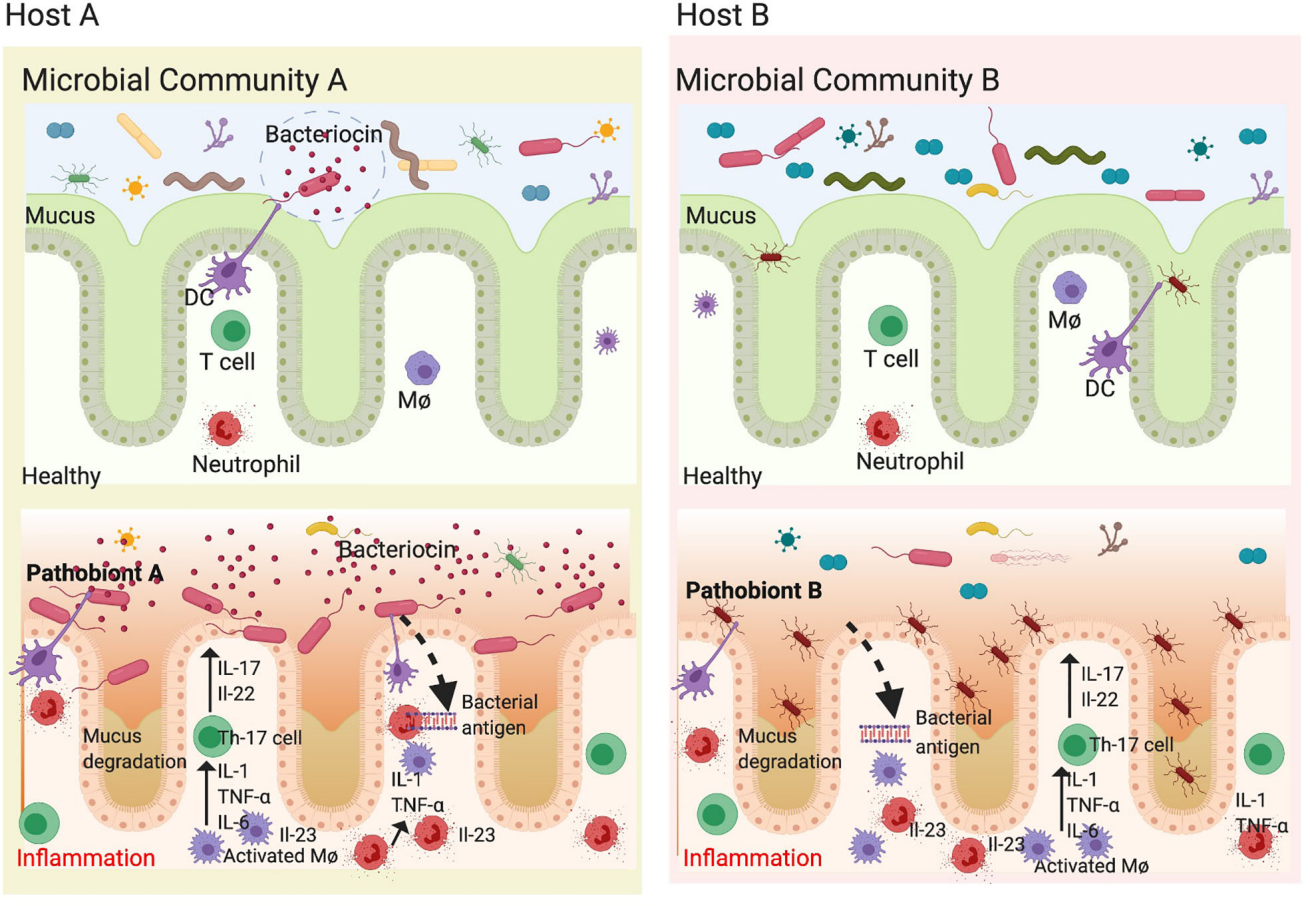
Pathobionts depend on host genetics and gut microbial community. The gut microbiota is highly diverse and varies between healthy individuals depending on host genetics, diet and environment. Each individual microbial community consists mostly of commensals which provide colonization resistance to opportunistic pathobionts. In genetically susceptible individuals (HLA-B27), there is microbial dysbiosis concomitant to loss of epithelial barrier resulting in an inflammatory microenvironment which further increases the loss in commensal microbes. This presents a unique opportunity for pathobionts to thrive and exacerbate inflammation. Since different individuals have different microbial community structure and different pathobionts depending on their genetics and environment, this may explain why we observe different pathobionts associated with various spondyloarthropathies. In microbial community A, only pathobiont A is present and when the conditions change (a trigger). In addition, certain pathobionts can make bactericidal compounds known as bacteriocins to avoids colonization resistance. When the pathobiont A is in bloom, it increases and exacerbates inflammation by degrading mucus and disrupting epithelial barrier. However, in another microbial community on a distinct host genetic background and in the presence of pathobiont B, which is mucous associated, an increased relative abundance can be associated with disease as most microbes are unable to cross the mucous barrier. In both cases, the dendritic cells (DCs) and Macrophages (Mφ) release inflammatory mediators such as IL-23, TNF-α, and IL-6, which activate T helper 17 cells to make IL-17, IL-22 causing inflammation and disrupting epithelial barrier thereby perpetuating the inflammatory cycle. Figure created with Biorender.com.

Many microbes deemed as commensals can act as pathobionts under certain circumstances. In such cases, the pathogenicity of these pathobionts is dependent on the host genetics as well as the composition of the gut microbiome. In antibiotic treated mice, adding commensal *Bacteroides* spp. can induce the development of colitis in IBD-susceptible background, but not in IBD-non-susceptible background (144). This may explain why adding back commensal microbes such as ASF to germ-free HLA-B27 TG rats is sufficient to drive colitis as well as arthritis (35). Conversely, pathobionts have been shown to exert beneficial effects in certain disorders. As mentioned earlier, supplementation of *A. Muciniphila* in mice and humans has shown to improve various metabolic parameters in obesity (145, 146). These results suggest that a microbe can act as a pathobiont depending on the context of host genetics and their position in the microbial community structure (**Figure 3**). We reported that presence of segmented filamentous bacteria (SFB) in both Lewis and Fischer genetic backgrounds correlates with disease in the presence of HLA-B27 but not in wild type controls (12). SFB adheres to the epithelial cells in terminal ileum in rodents at the time of weaning and induces the development of Th17 cells (147). SFB is absent on the DA rat background and these animals are resistant to gut inflammation and arthritis in the presence of HLA-B27. In contrast to these studies, SFB was shown to protect against rotavirus infection and diarrheal disease (148). In another study, SFB was able to protect from *Citrobacter rodentium* induced colitis (147). This may suggest that the microbial community is complex, and the results from the mono-colonization studies, while important to determine mechanistic pathways, may not be sufficient to recapitulate fully functional microbial communities.

## Association vs Causation

The association between the host species and their symbiotic microbes is a result of millions of years of coevolution, which has resulted in a homeostatic balance between the gut microbial community in host health and disease (149, 150). While imbalance in the symbiotic microbial communities inhabiting our body has been linked to various SpAs (151), it is not clear whether microbial dysbiosis is the cause or the effect of these disorders. Experimental models of various SpAs can help elucidate the causal microbes and microbial pathways, which can be confirmed in patients. One such example is *Porphyromonas gingivalis*, a periodontal bacterium, which has been shown to colonize synovial joints and exacerbate collagen induced arthritis (152). This suggests that *P. gingivalis* plays a mechanistic role in arthritis due to its translocation to the joints.

A complication in determining causal microbial mechanisms is that host-microbiome interactions are complex and have a multitude of variables affecting them. This makes it challenging to determine the pathobiont or other microbial species responsible for the disease phenotype (153). While mono-colonization of germ-free animals is a simplistic tool for determining causation, it lacks complex inter-microbial interactions and may not recapitulate the complexities of a stable microbial community. Thus, instead of colonization with single commensal and/or pathobiont, development of complex model microbiomes or synthetic microbial communities (154) and better culture techniques to culture/characterize refractory microbes (155) may help determine disease mechanisms. Development of better animal models and/or methods to colonize selective microbes in a complex microbial community will enable us to directly approach the mechanisms and address causality (156).

## Disease Mechanisms

Determination of the causal relationships and the underlying mechanisms with which these microbes interact with each other and their host is vital to develop therapeutic targets. Deciphering the mechanisms of microbial function in health and disease is crucial to ascertain cause and result relationships. Pathobionts are thought to exert their effects on host physiology through mucin degradation, disruption of epithelial barrier function as well as the loss of colonization resistance by commensals, therefore making them foremost targets to investigate disease mechanisms.

### Mucin Degradation

The mucus layer provides the first layer of physical barrier by limiting the microbial contact with the host tissue. Mucin degradation by microbial metalloproteinases has been suggested to contribute to IBD pathogenesis (157). Pathobionts such as *Akkermansia* and *Prevotella*, which are implicated in HLA-B27-associated SpAs, are known to have mucin degrading capabilities albeit through different mechanisms. For example, *Prevotella* may contribute to inflammation by encoding enzymes (superoxide reductase and a phosphoadenosine phosphosulfate reductase), which enable *Prevotella* to resist host reactive oxygen species and outcompete microbes essential for mucosal homeostasis such as *Bacteroides* spp (68, 101); *Akkermansia* can exacerbate inflammation by degrading the mucus layer over epithelial cells, thereby weakening the epithelial barrier (158). Mucolytic bacteria could contribute to disruption of intestinal barrier and joint inflammation. One such bacteria, *R. gnavus* is known to express ß-glucuronidase, that can convert bile acids into inflammatory secondary bile acids (deoxycholic and lithocholic acids), associated with intestinal inflammation (78, 159).

### Mucosal Adherence and Barrier Disruption

Almost 60 percent of patients with HLA-B27-associated SpAs have microscopic gut lesions, with one third having overt gut inflammation (160). First degree relatives of SpA and IBD patients also show signs of subclinical gut inflammation and impaired gut epithelial barrier (161, 162). While the underlying mechanism is not fully understood, animal models of SpAs and IBD have highlighted the importance of gut mucosa for host-microbe interactions. Many pathobionts associated with HLA-B27 such as *Akkermansia*, *Prevotella* and *Mucispirillum* have the ability to adhere and degrade the mucus layer (11, 12, 101). When epithelial barrier is disrupted, gut commensal microbes such as *Bacteroides vulgatus* are also sufficient to cause and perpetuate IBD in immunocompromized mice (163). During dysbiosis, increase in the pathobionts can degrade the mucus layer and may activate local inflammatory response, which could lead to disruption of the epithelial barrier. While we do not know if pathobionts cause barrier disruption, or if barrier disruption triggers pathobiont bloom, both events are related and critical to disease pathogenesis.

### Loss of Colonization Resistance

Of the many symbiotic functions performed by gut commensals, formation of stable microbial communities is perhaps the most important function, since it provides colonization resistance to infections and pathobiont expansion. The gut microbial community is dynamic with constant struggle for niche and resources between the gut commensals and the opportunistic pathobionts for energy, resources and niche. In a healthy individual, the gut commensals can either kill the pathobionts by production of bacteriocins, outcompete them for resources or activate the immune response to produce antimicrobial peptides (164). One such mechanism is the fucosylation of epithelial cells that promotes colonization by commensals and resistance to pathogens (165). Under eubiosis, the host immune system has the ability to distinguish gut commensals from pathobionts, although the mechanisms are not clear. In a recent study, the authors demonstrate that pathobionts such as *Citrobacter rodentium* can trigger inflammatory Th17 cells, while gut commensals like SFB trigger tissue-resident homeostatic Th17 cells (166). These tissue resident Th17 cells do not make inflammatory cytokines or participate in inflammatory reactions and have a slow metabolism. On the contrary, Th17 cells in response to *C. rodentium* have an inflammatory effector potential (166). During dysbiosis, mucosa associated pathobiont/s can expand at the expense of gut commensal microbes, degrade mucus and activate host inflammatory response (11).

## Therapeutic Implications

Gut microbe/s or microbial metabolites may provide novel treatment opportunities in HLA-B27-associated SpAs. These approaches aim to answer the basic question- what parameters affect the reorganization of a stable microbial community after dysbiosis and/or inflammatory insult? Here we focus on the role of pre and probiotics, short chain fatty acid, diet and fecal microbiota transplant in alleviation or amelioration of the disease.

### Pre- and Pro-Biotics

Prebiotics and probiotics can contribute to maintaining healthy gut community and therefore play and important role in the overall health of the gastrointestinal tract (167). Prebiotics are plant-based fiber, which may enhance the activity of beneficial gut bacteria, thereby promoting host health (168). On the other hand, probiotics consists of live bacterial strains, which upon ingestion in adequate amounts may confer health benefit to the host (169). Studies on HLA-B27 TG rats treated with prebiotic compounds inulin and oligofructose, demonstrated reduced colitis severity, associated with increase in the relative abundance of *Lactobacillus* and *Bifidobacterium* species. Prebiotic treatment significantly decreased inflammatory cytokines such as IL-1β and increased the levels of TGF-β, an immunomodulatory cytokine in the cecal tissue of these rats (170). In another study by the same group, the prebiotic combination of inulin and oligofructose was also effective in partially preventing colitis. While the HLA-B27 TG rats showed an altered microbial community, concomitant with an increase in Bifidobacterium, they were unable to observe any changes to the luminal short chain fatty acid concentrations (171). A later study by another group tried to dissect the effects of prebiotic treatment by using either inulin or fructo-oligosaccharide in HLA-B27 TG. All of the HLA-B27 TG rats which were fed fructo-oligosaccharide showed significant reduction in colitis, whereas only half of the HLA-B27 TG rats fed inulin showed improvement in colitis. While both groups were associated with decrease in the *Clostridium cluster XI*, rats fed fructo-oligosaccharides showed increase in *Bifidobacterium*, and inulin fed HLA-B27 TG rats showed an increase in *Bacteroides, Prevotella*, *Porphyromonas* group (172). Another group also measured the impact of a probiotic and prebiotic combination (*Lactobacilli, Bifidobacteria*, and inulin) on the severity of colitis and microbial community in HLA-B27 TG rats. Colitis was attenuated in HLA-B27 rats which received the probiotic, and they showed increase in microbial diversity, specifically an increase in the *Bifidobacterium animalis* (173). These studies show the promise of prebiotic and probiotic supplements as therapy for gut inflammation associated with various SpAs by renewing and restoring host gut microbial communities.

### Short Chain Fatty Acids

Another important contribution of gut microbiota is the production of microbial metabolites such as butyrate, propionate, and acetate; collectively known as short chain fatty acids (SCFA). These are the primary products of non-digestible carbohydrates/prebiotics, and their oxidation provides a major source of energy for the colonocytes (174). Microbial dysbiosis is accompanied by perturbations in the microbial metabolic function including changes in production/oxidation of short chain fatty acids (SCFA) and trimethylamine N-oxide (TMAO), which play an important role in modulation of host physiology [reviewed in (175)]. Asquith and colleagues showed that HLA-B27 expression alters the host and microbial metabolic profile with an increase in the levels of histidine, tyrosine, spermidine, N-acetylmuramate and glycerate in HLA-B27 TG rats (140). When supplemented with propionate (a SCFA), HLA-B27 TG demonstrated attenuation in the inflammatory disease. In another study, administration of propionate was shown to attenuate the severity of uveitis in an inducible model of experimental autoimmune uveitis (176). Propionate and other SCFAs such as butyrate can activate G protein-coupled receptors, GPR41 and GPR43 (177) (124). Mice deficient in GRP43 show exacerbation of inflammation in colitis, arthritis and asthma. The authors demonstrated that resolution of inflammatory response was dependent on the activation of GPR43 by SCFA (178). SCFAs such as butyrate have shown to effect the host innate immune function (179). They showed a reduction in inflammatory cytokines produced by macrophages *in vitro* when treated with butyrate, as well as in macrophages isolated from mice whose drinking water was supplemented with butyrate. Taken together, SCFAs play an important role in mucosal homeostasis by not only fueling the colonocytes, but also by suppressing the innate immune cells from mounting an inflammatory response. These diverse roles highlight the importance SCFAs as a therapeutic in various inflammatory (SpA, IBD) as well as metabolic (type I diabetes) diseases.

### Diet

Diet plays a major role in microbial community maintenance since it is an important source of small molecules, which are converted to various metabolic products by gut bacteria. High fat westernized diet is thought to be associated with various inflammatory disorders, whereas high fiber diet is associated with amelioration of inflammation (180). In a study by Rodrigues-Cabezas and colleagues, amelioration of colitis was reported in HLA-B27 TG rats given fiber enriched diet (181). This was associated with increased production of SCFA (butyrate and propionate), which in turn acted synergistically to inhibit pro-inflammatory mediators (181). Supplementing the diet with butyrate in mice has been shown to mediate the homeostasis of regulatory T cells (182, 183). In a mouse model, butyrate supplementation increased serotonin derived activation of the aryl hydrocarbon receptor (AhR). AhR is a transcription factor involved in the sensing of environmental signals like the redox potential (184); and recognized as the mediator for various ligands from diet, commensal microbes and host metabolites [Reviewed in (185)]. While AhR has recently been discovered as a B cell transcription factor and induces the development of regulatory B cells (186), its effect on immune modulation has been shown in IBD. Indole derivatives from cruciferous vegetables activate the AhR, and plays an important role in the maintenance of innate lymphoid and intraepithelial lymphocytes (187, 188). AhR receptors are expressed by peripherally derived regulatory T cells in the gut, and their expression has been shown to play a key role in the gut homing and anti-inflammatory functions of gut regulatory T cells (189). They are known to be involved in the maintenance of epithelial barrier and dampen various inflammatory conditions (190, 191). A study by Maslowski et al. (178), showed that decreased intake of complex plant polysaccharide fiber perturbs the microbial community leading to decreased production of SCFAs. Thus, diet has a role in maintenance of gut microbial community, which promotes gut health by increasing host beneficial metabolites as well as by maintaining community resistance by gut commensals to pathogenic microbes.

### Fecal Microbiota Transplant (FMT)

The role of microbiota in disease pathogenesis and evidence from FMT for *Clostridium difficile* infection makes FMT a promising treatment for HLA-B27 associated spondyloarthropathies. While FMTs are currently not available for spondyloarthropathies, FMT trials are underway in patients with RA (ClinicalTrials.gov identifier: NCT03944096), who are refractory to methotrexate treatment, as well as in a European cohort for psoriatic arthritis (ClinicalTrials.gov identifier: NCT03058900). Trials are also underway for the FMT treatment of AS patients (ASGUT- ClinicalTrials.gov Identifier: NCT03726645). In a recent report by (192), FMT treatment for *C. difficile* infection resulted in a decrease in disease activity for PsA. Recently, FMT has been proven to be effective for the induction of clinical remission associated with endoscopic improvement in active ulcerative colitis concomitant with persistent increase in microbial diversity (193). A major concern with FMT trials is development of a standardized fecal sample, and the concerns for long term colonization of a foreign microbial community in a different host microenvironment. While patients with *C. difficile* pseudomembranous colitis offer very little colonization resistance to FMT, in other inflammatory diseases the resident microbial community can prevent the colonization of the microbial community from FMT. To standardize the gut microbiota for FMT, researchers have developed methods to develop a synthetic microbial community which mimics the fecal microbiota (154). This synthetic microbial FMT is under trials for *C. difficile* infection (Clinical trial registration number: CinicalTrials.gov NCT01372943). Together, these studies show a remarkable role of FMT in repopulating the gut microbiota and its role in disease amelioration.

## Conclusions and Future Directions

HLA-B27 has been recognized to associate with various SpAs, especially AS for almost 50 years. With the advent of culture free sequencing techniques, the role of microbiome and microbial dysbiosis in disease pathogenesis is now well accepted. However, we are beginning to appreciate the complexity of gut microbial communities which consist of bacteria, archaea, viruses, and fungi. If we include the inter-kingdom interactions as well the host-microbiota relationships as factors affecting the role of individual microbes, these interactions in a stable microbial community become very complex. However, spatial differences in microbial community structure along with difference due to host, environmental, and technological factors should be considered before we cement the role of certain microbes and their functions in HLA-B27 associated SpAs.

The emerging picture suggests an important role of pathobionts in contributing toward HLA-B27 associated SpAs. Mechanistic studies on these pathobionts in germ-free and gnotobiotic rodent models have provided fundamental insights into the role of microbes in disease pathogenesis. While this review has focused on bacteria and fungi in SpA and associated disorders, studies of viruses especially bacteriophages in the gut mucosal environment could help explain whether viruses play a role in development of SpA in genetically susceptible hosts. However, the gut microbiome is a complex ecosystem of polymicrobial communities and therefore understanding the functional/metabolic implications of these microbial perturbations in context of an established gut microbial community is of immense value in determining host-microbe causal relations in SpA. The mechanistic effect of pathobiont expansion and dysbiosis in the context of host genetics and environment, will provide opportunities to develop novel therapeutic targets for disease alleviation/amelioration in HLA-B27 associated SpAs.

## Author Contributions

TG and JR jointly created the review outline. TG wrote the review and JR provided editing. All authors contributed to the article and approved the submitted version.

## Funding

This project was supported by NIH Grant RO1 EY029266 and EY026572. JTR also receives support from the Grandmaison Fund for Autoimmunity Research, the Stan and Madelle Rosenfeld Family Trust, the William and Mary Bauman Family Foundation, Research to Prevent Blindness, and NIH Grant, P30 EY010572. TG is a Jane Bruckel early career investigator supported by Spondylitis Association of America.

## Conflict of Interest

JTR is an unpaid advisor to Viome.

The remaining author declares that the research was conducted in the absence of any commercial or financial relationships that could be construed as a potential conflict of interest.
